# Out of the blue: The first record of the genus *Heremites* Gray, 1845 (Squamata, Scincidae) from Pakistan

**DOI:** 10.3897/zookeys.1039.64146

**Published:** 2021-05-20

**Authors:** Rafaqat Masroor, Muhammad Idrees, Muhammad Khisroon, Qaisar Jamal, Daniel Jablonski

**Affiliations:** 1 Zoological Sciences Division, Pakistan Museum of Natural History, Garden Avenue, Shakarparian, Islamabad-44000, Pakistan Pakistan Museum of Natural History Islamabad Pakistan; 2 Department of Zoology, University of Peshawar, Peshawar, Pakistan University of Peshawar Peshawar Pakistan; 3 Department of Zoology, Comenius University in Bratislava, Ilkovičova 6, Mlynská dolina, 842 15 Bratislava, Slovakia Comenius University Bratislava Slovakia

**Keywords:** Afghanistan, Central Asia, *
Mabuya
*, morphology, range extension, taxonomy, *
Trachylepis
*

## Abstract

The genus *Heremites* Gray, 1845 is endemic to the Western Palearctic region, containing morphologically similar species with a not well resolved taxonomy. The genus has a broad distribution from North Africa to Central Asia, with the only known record from northeastern Afghanistan. Three species are currently recognized in the genus with one, *H. septemtaeniatus* (Reuss, 1834), representing populations at the eastern edge of the genus range. During extensive fieldwork, we discovered *H. septemtaeniatus* from northwestern Pakistan and provisionally suggest that this population could be morphologically defined as *H. septemtaeniatus transcaucasicus* (Chernov, 1926). This important contribution to the knowledge regarding the family Scincidae in Pakistan, however, needs further investigation using an integrative approach.

## Introduction

**Figure 1.**
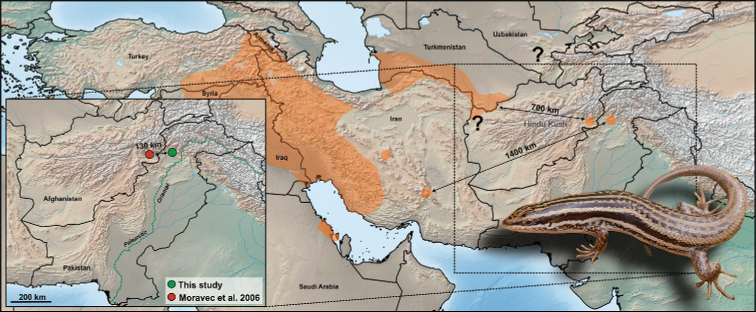
Distribution range of *Heremites septemtaeniatus* highlighted in orange, adopted from Sindaco and Jeremčenko (2008) and Šmíd et al. (2014), and species records from Afghanistan and Pakistan (Moravec et al. 2006; this study). The question marks indicate areas of questionable occurrence of the species from Afghanistan and Uzbekistan (Sindaco and Jeremčenko 2008). The pictured live specimen (DJ 9560) is an individual from Shah Alam Baba, Tehsil Adinzai, Lower Dir district, Khyber Pakhtunkhwa Province, Pakistan.

The endemic western Palearctic genus *Heremites* Gray, 1845 was recently resurrected and used for the revised taxonomy of the Middle Eastern lizard members of the *Mabuya* group (Karin et al. 2016). For a long time, members of the present-day genus *Heremites* were lumped into the genus *Trachylepis* Fitzinger, 1843, which now represents only related lizards from Africa and Madagascar (Karin et al. 2016). Based on the results of molecular phylogenetic analyses and morphology, the genus *Heremites* currently represents three species, *H. auratus* (Linnaeus, 1758), *H. septemtaeniatus* (Reuss, 1834), and *H. vittatus* (Olivier, 1804). They ranging from North Africa, through the Middle East and Arabia, to Central Asia (Sindaco and Jeremčenko 2008; Karin et al. 2016). However, this range is not, according to the current knowledge, connected, but is instead disjunct or further divided (Sindaco and Jeremčenko 2008). This range characteristic, together with molecular data suggest, that the current knowledge on these taxa is probably incomplete (cf. Baier et al. 2017; Bahmani et al. 2018). Morphological similarities between the three species and the unclear distribution throughout their range has led to confusion also in the taxonomy. Especially two species, *H. auratus* and *H. septemtaeniatus* are confusing despite their monophyletic position in molecular-based phylogenetic trees and the degree of genetic divergence (Mausfeld and Schmitz 2003; Güçlü et al. 2014; Karin et al. 2016; Bahmani et al. 2018). Some authors thus rank both taxa under the *H. auratus* species complex (Sindaco and Jeremčenko 2008). Moravec et al. (2006), however, restricted the range of *H. auratus* to Turkey and the adjacent Aegean Islands and mentioned *H. septemtaeniatus* as occurring in NE Africa, the Arabian Peninsula, Transcaucasia, Iraq, Iran, and western and southern Turkmenistan (Sindaco and Jeremčenko 2008; Fig. 1). Moravec et al. (2006) also reported *H. septemtaeniatus* for the first time from Afghanistan (Nangarhar Province), although Leviton and Anderson (1970) mentioned possible records from the vicinity of Tajan River at the Iran-Afghan-Turkmen borders (see also Sindaco and Jeremčenko 2008). The only known population of *H. septemtaeniatus* in Afghanistan is a biogeographical mystery, as it is separated from the rest of the genus range by the important barrier (the Hindu Kush Mountains) and desert basins (Sistan). The information presented in Moravec et al. (2006) represents a very important range extension for the genus, with a distribution gap of about 1300 km as the crow flies from localities in central Iran (Šmíd et al. 2014), and ca. 700 km from expected localities in NW Afghanistan or southern Turkmenistan (Sindaco and Jeremčenko 2008; Fig. 1 in this study). The origin of this isolated Afghan population is unknown and it has not been studied. Therefore, the biogeography and possible taxonomical consequences of the isolated eastern Afghan population remain challenging.

According to the current knowledge, the range of *H. septemtaeniatus* from Armenia, northern Iraq through central and northern Iran to eastern Afghanistan represents a subspecies, *H. s. transcaucasicus* (Chernov, 1926). Although the taxonomic status of the subspecies transcaucasicus is not clear and some authors refer to it as a subspecies of *H. auratus* (e.g. Leviton et al. 1992; Anderson 1999; Faizi and Rastegar-Pouyani 2006; Rastegar-Pouyani et al. 2008; Faizi et al. 2010; Bahamani et al. 2016; Karin et al. 2016; Bahamani et al. 2018), we here follow Achmedov and Ščerbak (1987) and Moravec et al. (2006) in recognizing it as a subspecies of *H. septemtaeniatus*. However, as is evident from recent molecular studies, the situation is probably more complex (Bahmani et al. 2018) and further molecular and morphological research across the range of the genus is required to resolve the taxonomic ambiguities.

The knowledge of reptile diversity in Pakistan has strikingly increased within the last two decades (e.g., Khan et al. 2021). On the other hand, the species composition, distribution patterns, and natural history of many species are still poorly known (Khan 2006; Masroor 2012). Following by Minton (1966), Khan (2006) adopted Murray (1884) in presenting the record of *Mabuya aurata* (= *H. auratus*) from Sindh, Pakistan as uncertain and dubious. This record is probably an error and the genus *Heremites* had thus previously never been recorded in the country, certainly not in the Sindh Province, which does not offer suitable climatic and habitat preferences for this genus. However, in view of the presence of the genus in one known Afghan locality in the Nangarhar Province (Moravec et al. 2006), distanced several tens of kilometers from the Pakistani border, we expected the possible presence of this genus in Pakistan.

## Material and methods

During field surveys in the Khyber Pakhtunkhwa Province, Pakistan, we found a population of lizards of the family Scincidae, identified as members of the genus *Heremites*. Overall, 13 specimens (seven adult males and five adult females [SVL (snout-vent length) ≥ 50 mm], and one juvenile) were collected during several trips between 2013 and 2019. All the specimens were caught by hand, euthanized in a closed vessel with a piece of cotton wool containing ethyl acetate (Simmons 2002) and later permanently fixed in formaldehyde or 70% ethanol. Specimens were deposited in the herpetological collection of the Pakistan Museum of Natural History (**PMNH**) in Islamabad, Pakistan, except one that is in the herpetological collection of the Department of Zoology, Comenius University in Bratislava, Slovakia as (DJ [Daniel Jablonski] 9560).

Morphological characters were taken following the character definitions by Faizi and Rastegar-Pouyani (2006) and Faizi et al. (2010). Measurements were taken with a digital caliper to the nearest 0.1 mm. Measurements of arms, legs, and head were principally taken on the right side of the animal (from the left side if the animal was damaged on the right). Scale counts beneath the fourth toe and finger was taken from both sides. Morphometric characters and scalation data were taken as follows: SVL (snout-vent length, from the tip of snout to the anterior edge of the cloaca); TL (tail length, from the posterior edge of the cloaca to the tip of the tail); HL (head length, the distance between the retroarticular process of the jaw and the snout-tip); HW (head width, the widest part of the head); HH (head height, from the occiput to the underside of the jaws); TrL (trunk length, distance from axilla to groin measured from the posterior edge of the forelimb insertion to the anterior edge of the hindlimb insertion); OD (orbital diameter, the vertical diameter of the orbit); EL (ear length, the longest dimension of the ear opening); DN (distance between nostrils); END (eye-nostril distance, the distance between the anterior corner of the eye and the tip of the snout); EED (eye-ear distance, from the posterior edge of the eye to the anterior corner of the ear); FrW (frontal width); FrL (frontal length); FnW (frontonasal width); FnL (frontonasal length); LorWa (width of anterior loreal); LorWp (width of posterior loreal); IpL (length of interparietal); MnW (width of mental); MnL (length of mental); HLL (hindlimb length, length of femur and crus to tip of fourth toe); FLL (forelimb length, length of humerus and forearm to tip of fourth finger); SL (supralabials); IL (infralabials); SSLE (number of scales between last supralabial and ear opening); EP (ear pectination, number of scales projecting inside the ear opening); PN (pair of nuchals); SAB (scales across the body, number of scales in a single row around the widest part of the body); DSNV (dorsal scales in a row from first nuchal to above level of the vent); VT (ventral transverse, scales counted in a row from chin shields to cloaca); SDL 4^th^T (subdigital lamellae under 4^th^ toe); SDL 4^th^F (subdigital lamellae under 4^th^ finger); PN (pair of nuchal scales); SCS (number of supraciliary scales); SC (number of subcaudals from behind vent to tip of tail). Qualitative characters: SOF (contact between the third supraocular and the frontal); PFC (prefrontals in contact or not); PSC (parietal shields in contact or not); KDS (number of keels on dorsal scales); in contact (+), without contact (-). The following data are presented as the ratio between obtained characters: SVL/TL, HL/HW, HW/HH, SVL/TrL, FrW/FrL, FnW/FnL, LorWa/LorWp, MnW/MnL.

For comparison of morphological data, we used data from adult specimens (n = 61), comprised of 48 specimens (belonging to *H. septemtaeniatus transcaucasicus* and *H. s. septemtaeniatus*) from Iran (Faizi and Rastegar-Pouyani 2006), and a single known specimen from Afghanistan (Table 1; Appendix 1). The single museum specimen (adult male, Figs 3 and 5) referred to *H. septemtaeniatus* from Afghanistan (ZFMK-H 9064) was collected on 7 April 1972 in the vicinity of Dar-e-Nur, vic. Shewa (34.5558°N, 70.6073°E), Nangarhar Province (Moravec et al. 2006; Wagner et al. 2016). This specimen represents the nearest record of the species to the presently described specimens of the genus *Heremites* from Pakistan. Morphological data from the Afghan specimen were taken *de novo* in this study and are presented here for the first time (Table 1). The distribution map was prepared using QGIS (2021). Institutional abbreviations for the voucher specimens are as follow: **ZFMK**: Zoologisches Forschungsmuseum Alexander Koenig, Bonn, Germany; **PMNH**: Pakistan Museum of Natural History, Islamabad, Pakistan; **RUZM**: Razi University Zoological Museum; **MMTT**: Iran National Natural History Museum; **DJ**: Daniel Jablonski (collection at the Department of Zoology, Comenius University in Bratislava, Slovakia).

**Table 1.** Data comparison of morphological characters of adults (minimum-maximum with mean ± standard deviation) of *Heremites septemtaeniatus* from Pakistan and Afghanistan with those of closely related taxa from Iran (Faizi and Rastegar-Pouyani 2006). All measurements are in mm, abbreviations are defined in the materials and methods section; (NA) data not available.

Characters	*Heremites septemtaeniatus*	*Heremites septemtaeniatus*	*Heremites s. transcaucasicus*	*Heremitess. septemtaeniatus* Iran
	Pakistan	Afghanistan	Iran	
	n = 12	n = 1	n = 39	n = 9
**Metric data**				
SVL	51.7–92.3 (82.9±6.9)	92.0	71.2–96.5 (81.8±6.0)	64.0–84.8 (74.6±6.9)
TL (complete tail n = 7)	68.0–111.7 (97.0±15.7)	105.8	72.0–129.0 (100.5±10.9)	89.7–160.0 (112.6±30.5)
SVL/TL (n = 7)	0.6–1.0	0.9	0.7–1.2	0.5–0.9
HL	11.1–19.0 (16.4±2.2)	16.5	NA	12.1–15.5 (13.7±1.1)
HW	8.2–11.0 (9.7±0.9)	11.0	NA	9.3–12.1 (10.7±0.8)
HL/HW	1.6–1.8	1.5	0.8–1.3	1.2–1.4
HW/HH	1.2–1.4	1.3	1.1–1.5	1.1–1.4
HH	5.8–8.7 (7.5±1.1)	8.1	NA	7.0–9.8 (8.4±0.9)
OD	1.8–3.0 (2.6±0.3)	2.8	1.1–2.8 (1.9±0.4)	1.5–2.1 (1.8±0.2)
EL	1.3–2.4 (1.8±0.3)	2.2	NA	NA
END	3.5–5.1 (4.4±0.5)	4.1	3.3–5.2 (4.2±0.5)	3.4–4.7 (4.1±0.5)
EED	3.6–5.6 (4.9±0.6)	5.0	4.1–5.9 (5.2±0.4)	5.0–6.1(4.9±0.5)
DN	1.7–3.0 (2.2±0.4)	2.5	1.9–3.6 (2.7±0.4)	2.1–3.0 (2.5±0.2)
HLL	24.3–36.1 (31.5±3.9)	35.0	NA	33.7–42.0 (38.6±3.0)
FLL	16.0–26.3 (22.4±2.9)	25.1	NA	21.5–28.0 (25.8±1.9)
TrL	22.8–46.8 (37.0±7.9)	49.1	33.0–51.6 (39.2±4.4)	27.7–40.5 (35.5±4.7)
SVL/TrL	1.8–2.3 (2.0±0.1)	1.9	1.8–2.4	1.9–2.3
FrW/FrL	0.5–0.6 (0.6±0.0)	0.5	NA	NA
FnW/FnL	1.4–2.0 (1.7±0.2)	1.6	NA	NA
LorWa/LorWp	0.5–1.0 (0.7±0.1)	0.5	NA	NA
IpL	2.1–3.0 (2.5±0.0)	3.6	NA	NA
MnW/MnL	1.6–2.0 (1.9±0.1)	1.5	NA	NA
**Meristic data**				
SL	6–7 (6.9±0.3)	6	NA	NA
IL	7–8 (7.0±0.3)	8	NA	NA
SSLE	3–4 (3.7±0.0)	4	NA	NA
EP	3–5 (4.0±0.6)	5	NA	NA
SAB	35–42 (36.0±2.0)	35	32–40 (36±1.8)	31–36 (32.9±1.5)
DSNV	52–60 (54.8±2.4)	54	NA	NA
VT	62–69 (65.0±2.2)	62	61–72 (66.6±2.8)	64–70 (67.1±2.0)
SDL 4^th^T	19–23 (21.0±1.1)	19–21	16–22 (18.7±1.6)	14–20 (17.5±1.8)
SDL 4^th^F	14–16 (14.8±0.7)	10–15	11–16 (14±1.1)	14–21 (15.6±2.2)
PN	1	1	NA	NA
SCS	5	4	NA	NA
SC (n = 7)	72–98 (85.8±9.0)	86	NA	NA
SOF	+	+	NA	NA
PSC	+	+	NA	NA
PFC	9(+), 3 (-)	+	NA	NA
KDS	3	3	3	3

## Results

**Figure 2.**
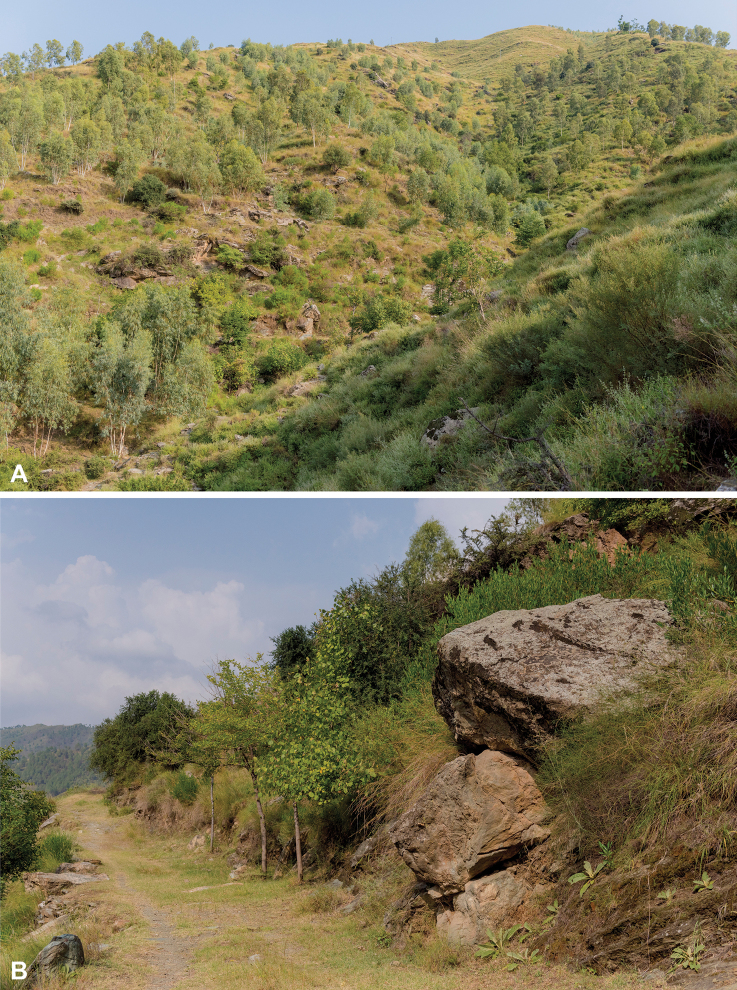
The habitat of *Heremites septemtaeniatus transcaucasicus* near Shah Alam Baba, Tehsil Adinzai, Lower Dir district, Khyber Pakhtunkhwa Province, Pakistan. Overall view of the locality (**A**), detail of the microhabitat (**B**).

**Figure 3.**
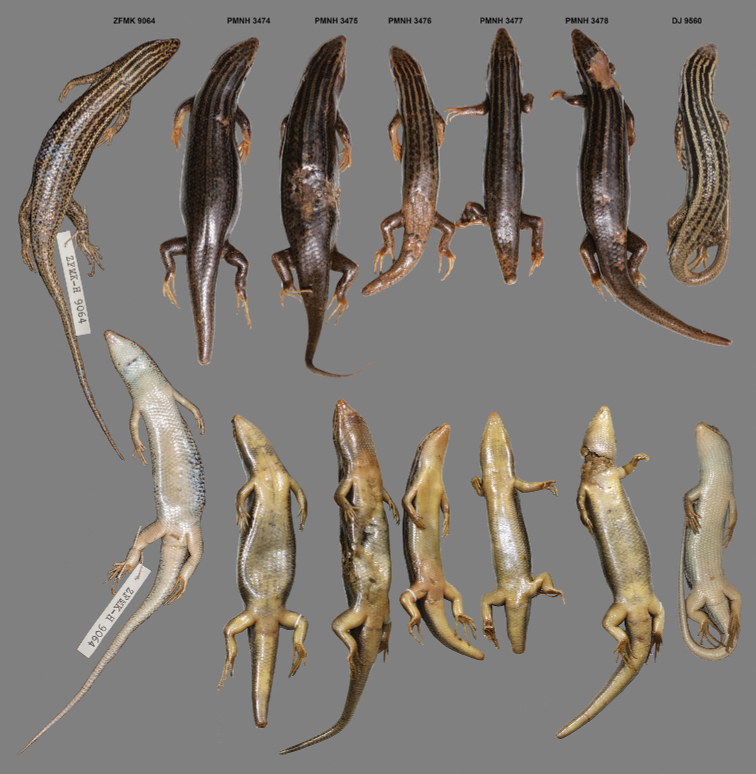
Dorsal and ventral views of adult specimens of *Heremites septemtaeniatus transcaucasicus* from Pakistan (PMNH 3474–3478 and DJ 9560), together with the only specimen of this species from Afghanistan (Dar-e-Nur, vic. Shewa, ZFMK-H 9064).

**Figure 4.**
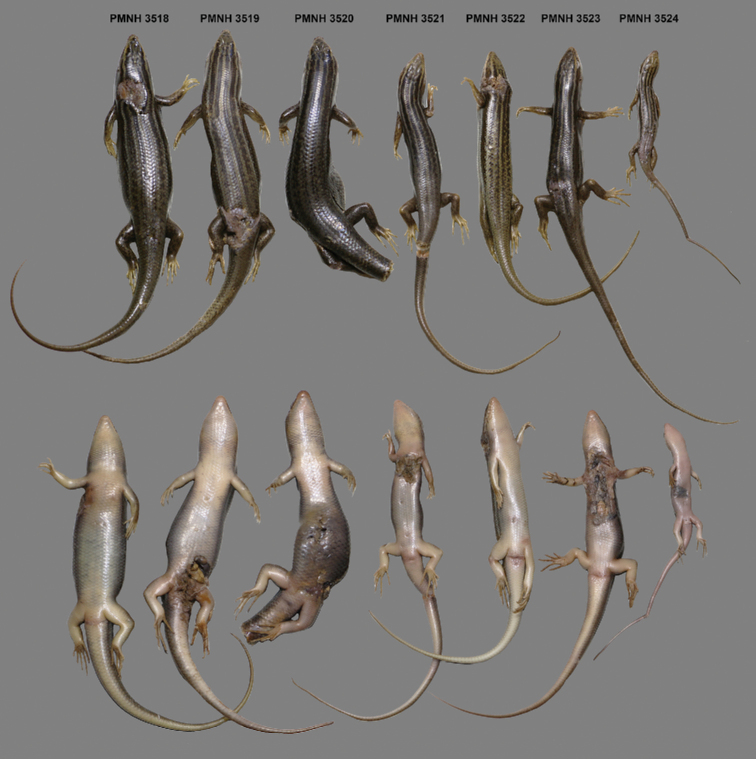
Dorsal and ventral views of adult and juvenile specimens of *Heremites septemtaeniatus transcaucasicus* from Pakistan (PMNH 3518–3524).

**Figure 5.**
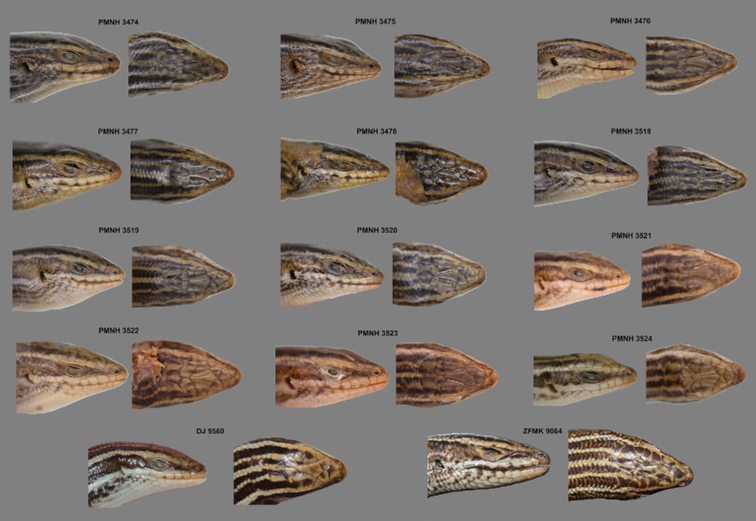
Lateral and dorsal head views of collected specimens of *Heremites septemtaeniatus transcaucasicus* from Pakistan (PMNH 3474–3478, PMNH 3518–3524 and DJ 9560), together with the only specimen of the species from Afghanistan (Dar-e-Nur, vic. Shewa, ZFMK-H 9064).

We report *Heremites septemtaeniatus* for the first time with certainty from the territory of Pakistan, representing the easternmost known distribution limit for the genus (Fig. 1). The first five individuals (PMNH 3474–3478; Fig. 3) were collected and morphologically identified as members of the genus *Heremites* during a field survey by M. Idrees on June 14, 2013. Seven additional specimens (PMNH 3518–3524; Fig. 4) were caught from the same locality on August 22, 2014. Very recently, another specimen (DJ 9560) was collected from the same locality on September 18, 2019 (Figs 1, 3, 5). The locality lies in the rocky habitat near Shah Alam Baba, Tehsil Adinzai, Lower Dir district, Khyber Pakhtunkhwa Province, Pakistan (34.7367°N, 72.1021°E; WGS 84, Fig. 2), at an elevation of 1110 m a.s.l. The collected specimens included both sexes and different age and size stages from juvenile to adults. This suggests that the population is well established and reproducing.

The region where the population was discovered is in the Lower Dir district, which has an average elevation of 1420 m a.s.l. The district is bestowed with three different forest types, i.e., moist temperate, sub-tropical Chir Pine, and sub-tropical broad-leaved. The elevation decreases gradually toward the south along the river Panjkora. The district lies in the temperate zone, where winters are cold with temperatures reaching below the freezing point (-2 °C), while summers are hot and humid due to heavy monsoon rains and with temperature reaching up to 32 °C (Nasrullah et al. 2012; Hidayat et al. 2017). The winter season arrives from mid-November to March and snowfall occurs in the upper parts from December to March. The investigated locality is a hilly area with big rocks that provided basking surfaces and shelter for the observed individuals of *Heremites*. Some of the frequently occurring trees in the study area were *Monotheca buxifolia*, *Eucalyptus camaldulensis*, *Ficus carica*, *Ailanthus altissima*, *Olea ferruginea*, *Morus alba* and *M. nigra*. The most dominant shrubs of the study area were *Dodonaea viscosa*, *Rumex hastatis*, and *Indigofera heterantha*. *Apluda mutica* was the most abundant grass species, followed by *Aristida depressa*, *Setaria viridis*, *Cymbopogan jwarancusa* and *Cynodon dactylon*.

The representative syntopic herpetofauna of the study area was documented and included amphibians [*Allopaa hazarensis* (Dubois & Khan, 1979), *Duttaphrynus melanostictus* (Schneider, 1799), *Firouzophrynus stomaticus* (Lütken, 1864), *Fejervarya* Bolkay, 1915 sp., *Hoplobatrachus tigerinus* (Daudin, 1802), Sphaerotheca cf. breviceps (Schneider, 1799)], lizards [*Calotes versicolor farooqi* Auffenberg & Rehmann, 1995, *Cyrtodactylus* (Gray, 1827) sp., *Eublepharis macularius* (Blyth, 1854), *Eurylepis taeniolatus* Blyth, 1854, Hemidactylus cf. brookii Gray, 1845, *Laudakia agrorensis* (Stoliczka, 1872), *L. pakistanica auffenbergi* Baig & Böhme, 1996, *Varanus bengalensis* (Daudin, 1802)], and snakes [*Boiga trigonata* (Schneider, 1802), *Bungarus caeruleus* (Schneider, 1801), *Echis carinatus sochureki* Stemmler, 1969, *Eryx johnii* (Russell, 1801), *Naja oxiana* (Eichwald, 1831), *Oligodon arnensis* (Shaw, 1802), *Ptyas mucosa* (Linnaeus, 1758), Platyceps cf. rhodorachis (Jan in de Filippi, 1865)].

The adult Pakistani specimens (n = 12) exhibited the following morphological characteristics (for details see Table 1): SVL 0.6–1.0 times TL; HL 1.6–1.8 times its width; HW 1.2–1. 4 times its height; SVL 1.8–2.3 times TrL; SVL in males (n = 7) 61.7–92.3 mm, in females (n = 5) 76.6–89.0 mm; TL in males 68.0–111.7 mm, 110.0 mm in females with complete tail; HL in males 11.1–19.0 mm, in females 16.4–18.0 mm; parietals in contact behind interparietal (100% of specimens); third supraocular in contact with the frontal (100%); prefrontals mostly in contact (75%) or separated (25%); 35–42 scales around the widest part of the body; 62–69 ventral scales in transverse rows counted from gular to cloaca; 52–60 dorsal scale rows from first nuchal to above vent; each dorsal scale provided with three keels; olive-brown above, with four longitudinal dark brown stripes on head dorsum, breaking up into rhomboidal spots towards the middle of the back and continuing up to tail base; broad dark stripe, bordered above with white spots, arising from the nostril, passing along upper half of the flank, continuing onto tail; limbs brown with white speckles (see Table 1).

Comparison of morphological data revealed that the Pakistani specimens are conspecific to specimen ZFMK-H 9064 from Afghanistan (Table 1, Figs 3 and 5). Except for slight variations in SCS, SDL 4^th^F, IL and IpL, the remaining morphological characters of the Pakistani specimens are in agreement with the Afghan specimen. On the other hand, specimens assigned to *H. septemtaeniatus* and *H. s. transcaucasicus* from Iran differ from the Pakistani specimens in several characters including HL/HW, HW/HH and SDL 4^th^T (Table 1). Based on the current knowledge, we are inclined to provisionally place the Pakistani specimens as *H. s. transcaucasicus*, pending further research. Our record of *H. septemtaeniatus* represents the 18^th^ taxon of the family Scincidae from the territory of Pakistan (Masroor 2012).

## Discussion

Information about geographic distributions are essential for understanding the biogeography, evolution, ecology of species and for enabling their effective conservation, especially at the margins of their ranges. Our first record of the genus *Heremites* for Pakistan extends the known range of the genus from the Afghan locality (see Moravec et al. 2006) by ca. 130 km as the crow flies to the east, ca. 700–800 km from localities in NW Afghanistan and Turkmenistan (Sindaco and Jeremčenko 2008), and ca. 1.400 km from the central Iranian localities (Anderson 1999; Šmíd et al. 2014; Fig. 1 in this study). The present Pakistani locality lies approximately only 70 km from the border between the Palearctic and Oriental biogeographical regions [see Palearctic-Oriental transition zone in Sindaco and Jeremcenko (2008; Fig. 1)]. Our record is thus a biogeographically very important range extension and another example of a West Palearctic reptile with a wide area of distribution reaching close proximity to the Oriental Region. For example, a similar pattern can be observed in *Laudakia caucasia* (Eichwald, 1831), *Natrix tessellata* (Laurenti, 1768), or *Macrovipera lebetinus* (Linnaeus, 1758) (Khan 2006; Sindaco and Jeremčenko 2008; Mebert et al. 2013; Sindaco et al. 2013; Jablonski and Masroor 2020). On the other hand, a similar distribution pattern can be observed in scincid lizards from the Oriental Region, as is well known for the genus *Eutropis* Fitzinger, 1843, which extends from SE Asia to Afghanistan (Karin et al. 2016; Wagner et al. 2016; Jablonski et al. 2019).

We suggest that the populations from Pakistan and Afghanistan should be tentatively ranked under *H. septemtaeniatus transcaucasicus* despite the fact that recent works (Faizi and Rastegar-Pouyani 2006; Rastegar-Pouyani et al. 2008; Arakelyan et al. 2011; Nasrabadi et al. 2017) treated the taxon *transcaucasicus* as a subspecies of *H. auratus*. Moravec et al. (2006) examined the type series of *H. septemtaeniatus* and *H. auratus* and pointed out differences in several morphological characters. These authors mentioned that the third supraocular is in contact with the frontal shield in *H. septemtaeniatus* while such contact is lacking in *H. auratus*. The third supraocular is also usually in contact with the frontal in *H. s. transcaucasicus* (Chernov 1926: 64) and thus, placement of *transcaucasicus* under the *H. septemtaeniatus* instead of *H. auratus* is justified. While describing *Mabuya transcaucasica*, Chernov (1926) did not provide information on the contact of the parietals. Nevertheless, the parietals are said to be in contact in *Heremites vittatus* while separated from each other in *H. septemtaeniatus* and *H. auratus* (Anderson 1999). Both *septemtaeniatus* and *transcaucasicus* exhibit an almost identical dorsal coloration and pattern by having four longitudinal dark brown stripes, breaking up into spots or disappearing on the posterior back. *Heremites auratus*, on the other hand, has two longitudinal rows of large, more or less rectangular dark spots on the dorsum. All specimens examined in the present study have parietals that are in narrow contact and thus, our specimens deviate in this character from what is typically characterized for the nominate species *septemtaeniatus* or its subspecies transcaucasicus (Anderson 1999: 274). Similarly, the subspecies transcaucasicus was described to have the prefrontals in contact so that the frontonasal does not come in contact with the frontal (Chernov 1926). On the contrary, the prefrontals are not in contact in *H. auratus* (Anderson 1999). Arrangement of prefrontals is, however, variable in the specimens at hand. In PMNH 3474–76, 3518–3519, 3520–3521, 3523–3524, the prefrontals are in contact and thus prevent the contact of the frontonasal with the frontal. On the other hand, PMNH 3522, 3477–3478, and ZFMK-H 9064 exhibit prefrontals which are separated so that frontonasal comes in contact with the frontal.

The disjunct distribution pattern of our *H. septemtaeniatus* together with its morphological differences from the known forms of the genus necessitate further research. It will be especially challenging to study DNA data to find out if the most probably isolated Hindu Kush population of *Heremites* has a relict distribution or even it belongs to an unknown taxon.

## Appendix 1

**Material used for the morphological comparison of *Heremites septemtaeniatus* from Iran (Faizi & Rastegar-Pouyani 2006).**

***Heremites s. transcaucasicus***

RUZM 001–005, West Azarbaijan Province, vicinity of Turkey border, Ghotur on the road between Lighwan and Sefideh khan (38.5833°N, 45.0333°E); RUZM 006–009, West Azarbaijan, Bukan, on the road to Mahabad (36.5333°N, 46.1666°E); RUZM 010–013, Kurdistan Province, Baneh, on the road to Saghez (35.9666°N, 35.9666°E); RUZM 014–020, Kurdistan Province, Marivan, on the road to Saghez (Sarshiv road), 35.3666°N, 45.2333°E); RUZM 021–029, Kurdistan Province, Sarvabad, (35.2833°N, 46.3500°E); RUZM 030–034, Kermanshah Province, on the road to Eslam Abad-e- Gharb, (34.3166°N, 47.1166°E); RUZM 035–039, Kermanshah Province, on the road to Paveh, Kawat, (34.9166°N, 46.4500°E).

***
Heremites **s. septemtaeniatus***

MMTT 1704, Khuzestan Province, Izeh; MMTT 1705, Khuzestan Province, East coast of Dez river; MMTT 1757, Khuzestan Province, Izeh; RUZM 050, Fars Province, Firouz Abad; MMTT 1841, Khuzestan, Ramhormoz; MMTT 1874, Khuzestan, Darkhuvin; MMTT 2116, Khuzestan, 20 Km S.W. Izeh; RUZM 051–52, Fars Province, Dashte Arjan.
